# Clinical and Genetic Correlates of Bipolar Disorder With Childhood-Onset Attention Deficit Disorder

**DOI:** 10.3389/fpsyt.2022.884217

**Published:** 2022-04-14

**Authors:** Nicolas A. Nunez, Brandon J. Coombes, Francisco Romo-Nava, David J. Bond, Jennifer Vande Voort, Paul E. Croarkin, Nicole Leibman, Manuel Gardea Resendez, Marin Veldic, Hannah Betcher, Balwinder Singh, Colin Colby, Alfredo Cuellar-Barboza, Miguel Prieto, Katherine M. Moore, Aysegul Ozerdem, Susan L. McElroy, Mark A. Frye, Joanna M. Biernacka

**Affiliations:** ^1^Department of Psychiatry & Psychology, Mayo Clinic, Rochester, MN, United States; ^2^Department of Quantitative Health Sciences, Mayo Clinic, Rochester, MN, United States; ^3^Lindner Center of HOPE /University of Cincinnati, Cincinnati, OH, United States; ^4^Department of Psychiatry & Behavioral Sciences, University of Minnesota Medical School, Minneapolis, MN, United States; ^5^Department of Psychiatry, Universidad Autónoma de Nuevo León, Monterrey, Mexico; ^6^Department of Psychiatry, Facultad de Medicina, Universidad de los Andes, Santiago, Chile

**Keywords:** ADHD, bipolar disorder, polygenic risk score, genetic, clinical features

## Abstract

**Background:**

Bipolar disorder (BD) with co-occurring attention deficit-hyperactivity disorder (ADHD) is associated with an unfavorable course of illness. We aimed to identify potential clinical and genetic correlates of BD with and without ADHD.

**Methods:**

Among patients with BD (*N* = 2,198) enrolled in the Mayo Clinic Bipolar Biobank we identified those with ADHD diagnosed in childhood (BD+cADHD; *N* = 350), those with adult-onset attention deficit symptoms (BD+aAD; *N* = 254), and those without ADHD (*N* = 1,594). We compared the groups using linear or logistic regression adjusting for age, sex, and recruitment site. For genotyped patients (*N* = 1,443), logistic regression was used to compare ADHD and BD polygenic risk scores (PRSs) between the BD groups, as well as to non-BD controls (*N* = 777).

**Results:**

Compared to the non-ADHD BD group, BD+cADHD patients were younger, more often men and had a greater number of co-occurring anxiety and substance use disorders (all *p* < 0.001). Additionally, BD+cADHD patients had poorer responses to lithium and lamotrigine (*p* = 0.005 and *p* = 0.007, respectively). In PRS analyses, all BD patient subsets had greater genetic risk for BD and ADHD when compared to non-BD controls (*p* < 0.001 in all comparisons). BD+cADHD patients had a higher ADHD-PRS than non-ADHD BD patients (*p* = 0.012). However, BD+aAD patients showed no evidence of higher ADHD-PRS than non-ADHD BD patients (*p* = 0.38).

**Conclusions:**

BD+cADHD was associated with a greater number of comorbidities and reduced response to mood stabilizing treatments. The higher ADHD PRS for the BD+cADHD group may reflect a greater influence of genetic factors on early presentation of ADHD symptoms.

## Background

Bipolar disorder (BD) is a severe episodic mood disorder with a considerable morbidity and premature mortality due to suicide and multiple medical comorbidities (1). Lifetime prevalence rate of BD is between 2.4 and 4.4 % (2, 3) and the comorbidity with attention deficit and hyperactivity disorder (ADHD) among adults diagnosed with BD has been estimated to range from 9 to 35% (4, 5) with a higher prevalence in BD type I (BD-I) (4). Importantly, the co-occurrence of BD and ADHD is associated with a significantly increased risk of addiction and anxiety disorders, which negatively impacts BD course of illness (4, 6, 7).

Diagnostic boundaries between BD and ADHD can been clinically difficult to discern due to overlapping symptoms and a persistence of ADHD from childhood to adulthood in certain cases. Moreover, longitudinal studies have shown that ~25% of individuals with childhood ADHD (cADHD) develop BD (8) although persistent attention deficits can also be linked to the natural course of BD illness. Although the symptomatic and syndromic overlap between BD and ADHD has been addressed extensively in the literature suggesting more of a mixed clinical presentation linked by the inattention domain, the neurobiological distinctive underpinnings of BD with and without comorbid cADHD remains unclear (9).

Both BD and ADHD have a substantial genetic component and thus many studies examining the overlap in BD and ADHD have focused on relatives of individuals with BD. Interestingly, a study on offsprings of BD patients considering high risk individuals, revealed there was no increase in ADHD diagnosis but a higher prevalence of hyperactive and mood/anxiety symptoms (10). Further, Meyer and colleagues found a higher prevalence of childhood attention and executive functions deficits and behavioral symptoms in the offsprings who developed BD compared to those with absence of a mood disorder in adulthood (11).

Polygenic risk scores (PRSs) are increasingly used in psychiatric studies because, in addition to estimating a person's genomic burden for a particular trait, they may demonstrate overlapping genetic predisposition between two traits (12). There have been previous studies that examined ADHD-PRS and its association with different psychiatric phenotypes. For example, a study examined the genetic risk for psychosis spectrum symptoms and different psychiatric phenotypes underscoring a significant association between ADHD-PRS and psychotic symptoms (13); also, a common genetic variation associated with risk for clinically diagnosed ADHD has been found to be associated with anxiety and depressive disorders amongst others (14). A recent study found that ADHD PRS was higher in BD patients diagnosed with cADHD compared to controls but was only marginally higher than in BD patients without cADHD (15). Moreover, a large Danish population study comparing genetic loci between childhood, persistent and late diagnosed patients with ADHD suggested a higher ADHD PRS was associated with persistent ADHD symptoms compared to cADHD or late ADHD (16).

Our aim in this study was to evaluate demographic, clinical, treatment, and genetic differences between BD with and without ADHD comorbidity. We extend this comparison to also consider the onset of attention deficits. Given the nosological controversies, we aimed to enhance the literature by replicating the previous findings of increased illness burden and associated sociodemographic features as well as treatment outcomes. Furthermore, we compared associations of genetic risk for ADHD and BD with the presence and time of onset of attention deficits.

## Materials and Methods

### Sample Description

The Mayo Clinic BD Biobank (MCBB) was established by a collaborative network with the aim of building a repository that will facilitate studies on disease risk, pharmacogenomics and treatment outcomes (17). Enrollment sites included: Mayo Clinic (Rochester, Minnesota), Lindner Center of HOPE/University of Cincinnati College of Medicine (Cincinnati, Ohio), University of Minnesota (Minneapolis, Minnesota), Clinica Alemana (Santiago, Chile) and Universidad Autonoma de Nuevo Leon (Monterrey, Mexico). Each site had its own protocol approved by the local Institutional Review Board, and all patients consented to use of their data for future genetic studies. Diagnostic confirmation of BD was determined using the Structured Clinical Interview (SCID) for DSM-IV (18). Mood disorder psychiatrists recorded clinical characteristics and current medications by review of all available clinical materials (i.e., electronic health record, patient interviews). Using the Clinical Questionnaire, clinicians recorded the presence or absence of current and/or lifetime diagnoses of ADHD during childhood (cADHD). We also explored the phenotype of attention deficits in adulthood (aAD) in patients with no cADHD diagnoses. For the genetic analysis, controls without BD were selected from the Mayo Clinic Biobank (19). Potential controls with International Classification of Disease-9 codes for BD or schizophrenia in their electronic medical record were excluded. Patients with other psychiatric illnesses (e.g., major depression) were not excluded.

### Clinical Measures

Patients were assessed for an anxiety disorder comorbidity domain (range 0–6) based on the sum of all lifetime anxiety disorders namely: post-traumatic stress disorder (PTSD), generalized anxiety disorder (GAD), social anxiety disorder (SAD), obsessive-compulsive disorder (OCD), phobia, and panic disorder. Similarly, we calculated a mood instability domain (range 0–5) which was determined by the sum of a lifetime presence of mixed episodes, rapid cycling, ultra rapid/ultradian cycling, cycle acceleration over time, and increased episode severity over time, being coded as no = 0 and yes = 1. Data related to family history, comorbidities and pharmacological treatments were collected at time of inclusion into the MCBB by clinical interview supplemented by information extracted from the electronic health records. Treatment response to lithium, antipsychotics and antiepileptic mood stabilizers (lamotrigine and divalproex) were assessed with the Alda scale (20). This scale was developed for retrospective evaluation of prophylactic treatment response in naturalistic conditions; it utilizes two subscales A and B. The A score is a composite measure of clinical improvement in severity, duration and frequency of illness and is rated from 0 (no change)-10 (complete response) thus a higher A score represents a greater improvement. The B score evaluates five potential confounders to determine the role of the medicine in improvement of BD: number of episodes before the treatment (B1), frequency of episode before the treatment (B2), duration of the treatment (B3), compliance during periods of stability (B4), and use of additional medications during the periods of stability (B5).

### Genotyping and Imputation for Overall Sample

A subset of the clinical sample had genotype data available. Genotyping and genetic data quality control of this sample were previously described (21, 22). Briefly, the Illumina HumanOmniExpress platform was used to genotype 1,046 BD cases and 828 controls. For quality control purposes, we excluded subjects with <98% call rate and related subjects (randomly choosing one individual in pairs with kinship coefficient > 0.2). SNPs with call rate <98%, MAF <0.01, and those not in Hardy-Weinberg Equilibrium (HWE; *P* < 1e-06 in healthy controls) were removed. After these steps 643 011 SNPs and 920 BD cases with ADHD assessment and 777 non-BD control subjects remained. Genotypes were imputed using the Michigan Imputation Server (23) with the HRC reference sample. Dosage data was converted to best guess genotype for the well-imputed (dosage R^2^ >0.8) and common (MAF >0.01) SNPs, resulting in more than 5 million SNPs.

### Polygenic Risk Scores

PRSs were calculated based on genome-wide association study (GWAS) summary statistics from the largest PGC studies of BD (24) and ADHD (25) and restricted to only well-imputed variants (INFO>0.9). Summary statistics removing the MCBB sample from Mullins et al. (24) were used to avoid sample overlap. LDpred2 (26) was used to compute the ADHD and the BD-PRSs using the “auto” setting which directly learns the two LDpred2 parameters from the GWAS data and creates one PRS for a given trait. PRSs were standardized to have standard deviation equal to one and centered with respect to controls such that controls have a mean of zero.

### Statistical Analysis

We used linear and logistic regression to compare demographic and clinical variables between BD+cADHD and non-ADHD (BD patients with no cADHD or aAD) groups. We also performed an exploratory analysis comparing these groups of patients to the BD+aAD group. All analyses were adjusted for age, sex and recruitment site. The PRS analyses also included comparisons with non-BD controls. For the PRS analyses logistic regression was used to predict each binary outcome (e.g., cADHD vs. non-BD controls) while adjusting for the first four principal components of ancestry (PCs) to control for population stratification. All statistical analyses were performed in R (version 3.5.1). To account for the multiple statistical tests in Table 1, we mainly highlight comparisons with *p* < 0.001, which approximately controls for the 30 variables compared between cADHD cases to non-ADHD BD cases.

**Table 1:** Comparison of demographic and clinical variables between BD patient subgroups: BD+cADHD, BD+aAD, and no-ADHD.

	**Child onset**	**No ADHD**	**cADHD**	**Adult onset**	**cADHD**	**aAD**
	**(cADHD)**	***N* = 1,594**	**vs No ADHD**	**(aAD)**	**vs aAD**	**vs No ADHD**
	**N=350**		**p-value**	***N* = 254**	***p*-value**	***p*-value**
Sex, Male, *N* (%)	174 (49.7%)	570 (35.8%)	<0.001^*^	89 (35.2%)	<0.001^*^	0.939
Age, Mean (SD)	34.9 (13.1)	43.0 (15.3)	<0.001^*^	42.5 (13.3)	<0.001^*^	0.707
Married (Y/N) *N* (%)	128 (37.9%)	720 (47.2%)	0.649	103 (41.7%)	0.209	0.185
Education > 12 years, *N* (%)	320 (94.7%)	1,458 (95.7%)	0.355	239 (96.8%)	0.616	0.533
Fulltime work, *N* (%)	72 (21.5%)	407 (27.2%)	0.002	56 (23.0%)	0.223	0.192
Diagnosis: BD-I vs. BD-II, *N* (%)	250 (71.4%)	1,080 (67.8%)	0.175	159 (62.6%)	0.077	0.130
Family history of bipolar disorder, *N* (%)	145 (52.9%)	541 (44.8%)	<0.001	107 (56.3%)	0.658	0.005
Family history of depression, *N* (%)	252 (87.5%)	1,043 (77.4.%)	<0.001	186 (86.9%)	0.307	0.001
Family history of alcoholism, *N* (%)	162 (54.5%)	639 (47.2%)	<0.001	119 (54.3%)	0.365	0.063
History of psychosis, *N* (%)	143 (41.4%)	621 (39.3%)	0.480	99 (39.3%)	0.736	0.910
History of suicidal attempts, *N* (%)	121 (35.1%)	528 (33.3%)	0.496	96 (37.9%)	0.536	0.171
History of rapid cycling, *N* (%)	163 (70.6%)	619 (59.7%)	0.037	114 (69.5%)	0.550	0.085
Nicotine dependence, *N* (%)	163 (47.4%)	595 (37.6%)	<0.001	106 (42.4%)	0.375	0.171
Alcohol dependence, *N* (%)	165 (48.1%)	541 (34.2%)	<0.001	104 (40.9%)	0.305	0.063
SUD (Cocaine- Methamphetamine)
*N* (%)	58 (17.0%)	32 (2.0%)	0.051	39 (15.5%)	0.564	0.218
Substance use disorder comorbidity sum
Mean (SD)	1.20 (1.05)	0.87 (0.98)	<0.001	1.05 (1.02)	0.211	0.020
Anorexia, *N* (%)	23 (6.7%)	76 (4.8%)	0.044	13 (5.2%)	0.218	0.698
Bulimia, *N* (%)	25 (7.3%)	85 (5.2%)	0.055	17 (6.7%)	0.454	0.334
Binge eating disorder, *N* (%)	44 (12.8%)	188 (11.8%)	0.465	37 (14.7%)	0.823	0.101
Generalized anxiety disorder, *N* (%)	201 (58.3%)	724 (44.5%)	<0.001	152 (61.3%)	0.812	<0.001
Social anxiety disorder, *N* (%)	105 (30.9%)	300 (18.4%)	<0.001	69 (27.5%)	0.475	<0.001
Panic disorder, *N* (%)	123 (35.7%)	440 (27.0%)	0.002	91 (36.3%)	0.476	0.007
Obsessive compulsive disorder, *N* (%)	56 (16.2%)	163 (10.0%)	0.005	63 (25.0%)	0.027	<0.001
Anxiety disorder comorbidity sum
Mean (SD)	1.7 (1.35)	1.3 (1.23)	<0.001	1.8 (1.4)	0.977	<0.001
Mood instability sum
Mean (SD)	1.8 (1.33)	1.5 (1.31)	0.017	1.88(1.4)	0.321	0.005
Alda A Scores, mean (SD)						
Antipsychotics	4.1 (2.5)	5.5 (2.8)	0.020	3.6 (2.8)	0.914	0.044
Divalproex	4.9 (2.6)	5.2 (2.8)	0.466	4.6 (2.2)	0.663	0.296
Lamotrigine	4.5 (2.5)	5.6 (2.5)	0.007	4.4 (2.6)	0.922	0.005
Lithium	4.7 (2.9)	5.6 (2.8)	0.005	4.4 (3.0)	0.761	<0.001
Adherence-Alda score B4	12 (12.1%)	77 (18.0%)	0.077	9 (13.4%)	0.910	0.048

*Comparisons are adjusted for age, sex, and recruitment site.*

*ADHD, attention deficit and hyperactivity disorder; SD, standard deviation; BD, bipolar disorder; BD-I, bipolar disorder type I; BD-II, bipolar disorder type II; SUD, stimulant use disorder; SCZ-BD, schizoaffective disorder bipolar type; Y/N, yes-no binary outcome.*

* *Non-adjusted by for age, sex and recruitment site*.

## Results

Table 1 summarizes demographic and clinical information of subjects with BD+cADHD (*n* = 350), non-ADHD (*n* = 1594), BD+aAD (*n* = 254) and comparisons between groups. Compared to non-ADHD, BD+cADHD patients were younger (34.9 ± 13.1 vs. 43.0 ± 15.3; *p* < 0.001), more likely to be men (49.7 vs. 35.8%; *p* < 0.001) and less likely to have full time employment (21.5 vs. 27.2%; *p* = 0.002). Similarly, comparing to those with aAD, BD+cADHD cases also were younger (34.9 ± 13.1 vs. 42.5 ± 13.3; *p* < 0.001), and more likely to be men (49.7 vs. 35.2%; *p* < 0.001). In comparing those with aAD vs. those with non-ADHD, there was no significant difference in gender (35.2 vs. 35.8%; *p* = 0.94) or in age (42.5 ± 13.3 vs. 43.0 ± 15.3; *p* = 0.71).

### Family History

BD+cADHD had greater prevalence of family history of mental illness: BD (52.9 vs. 44.8%, *p* < 0.001), depression (87.5 vs. 77.4%, p <0.001) and alcoholism (54.5 vs. 47.2%, *p* = 0.001) compared to non-ADHD. BD+aAD showed higher rates of BD (56.3 vs. 44.8%, *p* = 0.005) and depression (86.9 vs. 77.4%, *p* < 0.001) family history when compared to non-ADHD. There were no significant differences between BD+cADHD and BD+aAD in terms of family history.

### BD-Specific Clinical Presentation

BD+cADHD, BD+aAD and non-ADHD groups were not significantly different in regard to clinical presentation. They had similar rates of BD type I (71.062.6 and 67.8%, respectively), history of psychosis (41.4, 39.3, and 39.3%, respectively), suicide attempts (35.1, 37.9, and 33.3%, respectively), and rapid cycling (71, 70, and 60%, respectively).

### Anxiety Comorbidity

The BD+cADHD group exhibited a greater mean number of anxiety disorder comorbidities compared to non-ADHD (1.7 ± 1.3 vs. 1.3 ± 1.2, p <0.001) which included: greater lifetime prevalence of generalized anxiety disorder (58.3 vs. 44.5%; *p* < 0.001), social anxiety (30.9 vs. 18.4%; *p* < 0.001), panic disorder (35.7 vs. 27.0%; *p* = 0.002) and OCD (16.2 vs. 10.0%; *p* = 0.005). Similarly, the BD+aAD group had a greater overall number of anxiety disorder comorbidities compared to non-ADHD (1.8 ± 1.4 vs. 1.3 ± 1.2, *p* < 0.001) including generalized anxiety disorder (61.3 vs. 44.5%; *p* < 0.001), social anxiety (27.5 vs. 18.4%; *p* < 0.001), and OCD (25.0 vs. 10.0%; *p* < 0.001). The BD+aAD group did not differ from the BD+cADHD group with respect to anxiety comorbidity.

### Substance Use Comorbidity

BD+cADHD also had higher rates of overall substance use disorder comorbidities than non-ADHD (1.2 ± 1.0 vs. 0.87 ± 0.98, p <0.001) namely: alcohol dependence (48.1 vs. 34.2%, *p* < 0.001) and nicotine dependence (47.4 vs. 37.6%; *p* < 0.001). The BD+aAD cases also had slightly higher rates of overall substance use disorders than non-ADHD cases (1.05 ± 1.02 vs. 0.87 ± 0.98, *p* = 0.02) but did not differ from the BD+cADHD group.

### Pharmacological Treatment and Treatment Response

BD+cADHD and BD+aAD showed greater prevalence of stimulant use compared to non-ADHD (70.2 and 75.2%, respectively vs. 53.5%; *p* < 0.001), while BD+aAD had higher antidepressant use compared to non-ADHD (85.4 vs. 78.8%; *p* < 0.001) and BD+cADHD (85.4 vs 80.6%; *p* < 0.001). Treatment response assessed by the Alda scale showed that non-ADHD cases had significantly higher mean Alda A scores (better response) for lamotrigine (5.6 ± 2.5 vs. 4.5 ± 2.5, *p* = 0.007) and lithium (5.6 ± 2.8 vs. 4.7 ± 2.9, *p* = 0.005) compared to BD+cADHD. Similarly, non-ADHD cases had significantly higher mean Alda A scores for lamotrigine (5.6 ± 2.5 vs. 4.4 ± 2.6, *p* = 0.005) and lithium (5.6 ± 2.8 vs. 4.4 ± 3.0, *p* < 0.001) compared to BD+aAD. There were no differences in mean Alda A scores between the groups of BD patients with cADHD and aAD.

### Polygenic Risk Scores

Tables 2A,B summarize the associations of the ADHD and BD PRSs with groups and the proportion of variance explained by the PRSs (measured using Nagelkerke's pseudo-R^2^). All BD cases had higher ADHD PRS and BD PRS than controls regardless of ADHD subgroup (OR ≥ 1.26 for ADHD and OR ≥ 1.65 for BD; *p* < 0.001 for all comparisons). Further, BD+cADHD had a higher ADHD PRS than non-ADHD cases (OR = 1.20; *p* = 0.012) while there was no significant difference between those with aAD and without ADHD (OR = 1.07; *p* = 0.38). No other within-case comparisons were significant.

**Table 2A:** Associations of ADHD PRS with BD subtypes defined by cADHD and aAD.

**BD group**	***N***	**OR (95% CI)**	**R^**2**^**	***p*-value**	**Reference**
All	1,443	1.26 (1.15, 1.38)	1.5%	9.32E-07	No BD Control (*N* = 777)
No ADHD	963	1.23 (1.10, 1.36)	1.2%	7.28E-05	
cADHD	234	1.51 (1.28, 1.77)	4.0%	4.05E-07	
aAD	178	1.32 (1.11, 1.57)	1.7%	0.002	
cADHD	234	1.20(1.04, 1.38)	0.7%	0.012	BD no ADHD (*N* = 963)
aAD	178	1.07 (0.91, 1.26)	0.0%	0.38	
cADHD	234	1.12 (0.91, 1.36)	0.2%	0.26	BD+aAD (*N* = 178)

*ADHD, attention-deficit/hyperactivity disorder diagnosed during childhood (cADHD) or attention deficits diagnosed in adulthood (aAD); BD, bipolar disorder; OR, odds ratio; CI, confidence interval; PRS, polygenic risk score. R^2^-Nagelkerke's pseudo-R^2^*.

**Table 2B:** Associations of BD PRS with BD subtypes defined by cADHD and aAD.

**BD group**	**N**	**OR (95% CI)**	**R^**2**^**	***p*-value**	**Reference**
All	1,443	1.65 (1.49, 1.83)	6.4%	8.7E-23	No BD Control (*N* = 777)
No ADHD	963	1.70 (1.52, 1.90)	7.5%	1.3E-21	
cADHD	234	1.51 (1.27, 1.79)	3.6%	1.5E-06	
aAD	178	1.60 (1.32, 1.94)	4.0%	1.7E-06	
cADHD	234	0.90 (0.78, 1.04)	0.1%	0.188	BD no ADHD (*N* = 963)
aAD	178	0.91 (0.77, 1.07)	0.1%	0.273	
cADHD	234	0.98 (0.79, 1.21)	0.0%	0.884	BD+aAD (*N* = 178)

*ADHD, attention-deficit/hyperactivity disorder diagnosed during childhood (cADHD) or attention deficits diagnosed in adulthood (aAD); BD, bipolar disorder; OR, odds ratio; CI, confidence interval; PRS, polygenic risk score. R^2^-Nagelkerke's pseudo-R^2^*.

## Discussion

This study extends on previous studies by examining clinical features and two distinct PRSs (ADHD and BD) in a cohort of patients with BD and with or without ADHD by time of symptom onset, as well as a group of controls without BD. Our results are consistent with previous literature showing that attention deficits are more prevalent in men (27) and associated with lower rates of employment (28). In line with previous studies that reported a higher prevalence of ADHD in offspring of BD patients (29), BD+ ADHD patients showed significantly higher rates of family history of affective disorders and higher prevalence of substance use disorders. Specifically, we observed increased rates of alcohol use disorder and stimulants use, which is in accordance with earlier studies that reported a higher rate (reporting up to 44%) in ADHD patients of developing substance use disorders during their lifetime (30, 31). This underscores a potential worrisome consideration for clinicians as the use of stimulants may accelerate onset of BD (32) or contribute to the development of a future substance use disorder. However, a previous study showed that the use of stimulants during cADHD (ie. methylphenidate) was not associated with an increased risk to develop a later substance use disorder (33).

Our results suggest a higher prevalence of anxiety and depression disorders in patients with attentional deficits which is consistent with previous observations (29, 34); conversely, our findings differ from an earlier study that reported higher anxiety symptoms but not anxiety disorders (35). Despite focusing on the symptomatic overlap between disorders, anxiety disorders *per se* and ADHD symptoms have been linked to an overall worse psychosocial global functioning in pediatric BD patients (36) emphasizing the need for an accurate and early diagnosis to develop successful interventions.

Overall, our data showed that in terms of treatment response to mood stabilizers, the BD+cADHD group had a significantly poorer response to lithium and lamotrigine. These findings are in accordance with previous data where BD patients with attentional deficits generally exhibit worse outcomes (35). Interestingly there were no significant differences in treatment response by time of onset of attentional deficits.

Our findings also extend the literature showing a higher ADHD PRS in BD patients compared to controls (15). Grigoroiu-Serbanescu et al. (15) reported that ADHD PRSs tend to be higher in BD patients compared to healthy controls and showed marginally significant difference between BD patients with and without ADHD comorbidity. In our study we additionally observed that ADHD PRSs were higher in BD +cADHD compared non-ADHD BD cases, but not significantly higher when we compared to BD+aAD suggesting adult symptoms of inattention, without corresponding syndromal disorder in childhood, may simply be symptoms of bipolar disorder. Although the Grigoroiu-Serbanescu study had comparable sample sizes to ours (BD +cADHD = 365 and no-cADHD = 577) they tested PRS associations under multiple p-value thresholds requiring higher multiple testing corrections. Furthermore, our PRS results align with a recent study of ADHD patients from the iPSYCH cohort, which underscored a high genetic heterogeneity in ADHD subgroups with a higher polygenic risk load for childhood ADHD compared to late-onset ADHD (16).

To our knowledge, our study is the first to examine the BD PRS association with ADHD comorbidity in BD. However, we found no evidence that the genetic risk for BD was different between the different ADHD patients' subgroups. Additionally, while those with BD+ aAD have symptoms of inattention and receive stimulants at a much higher rate and longer duration of time than non-ADHD BD patients, we found no differences between the two groups in terms of ADHD genetic risk. Interestingly a significant difference was found for the BD+cADHD from non-ADHD, which could potentially suggest a genetic and phenotypic distinctiveness of the BD+aAD group between time of onset. These concepts of an intermediate phenotype were initially described by investigations of Post and colleagues regarding the nosological separation of ADHD and BD (37) emphasizing the intricacies underlying inattention deficits in adult BD patients. Furthermore, attentional deficits in adults with BD could be far more diagnostically related to core bipolar/soft hypo/manic and/or depressive symptoms.

Our findings should be considered in the context of several limitations. First, our data is extracted from a biobank with retrospective data collection and a relatively small sample size, particularly for attentional deficits with adult onset. Thus, our findings warrant replication in larger, prospectively assessed cohorts. Secondly, we did not have data regarding different ADHD subtypes, such as the inattentive, hyperactive or combined subtypes (38). Another limitation is that a structured clinical interview was used only to confirm BD diagnosis, while presence or absence of cADHD and aAD was based on patient interviews and the present information in the electronic health record. The use of a structured clinical interview or an operationalized rating scale (such as the Adult ADHD Self-Report Scale or the Wender-Reimherr Adult ADHD Rating Scale) to assess ADHD would have been preferred. Additionally, controls were not assessed for cADHD or aAD. However, potential controls with cADHD would only reduce the power of the PRS comparisons with controls rather than inducing false positive associations. Finally, our PRS analysis was restricted to patients with European ancestries, which may limit the generalizability of our findings to other populations.

Despite the aforementioned limitations, this work has broad relevance in terms of the nature of the relationship between ADHD and BD. It strengthens the hypothesis of a unique genetic pathophysiology in patients with BD+ADHD, particularly those with BD+cADHD, and differential response to certain mood stabilizers compared to non-ADHD patients. Improving our understanding of the clinical and genetic structure of this complex comorbid phenotype should improve diagnostic accuracy and design of future genetic and other biomarker studies leading to tailored interventions.

## Data Availability Statement

The data analyzed in this study is subject to the following licenses/restrictions: We used data from an existing biobank and a limited dataset is available upon request. Requests to access these datasets should be directed to frye.mark@mayo.edu; Website: https://www.mayo.edu/research/centers-programs/bipolar-disorder-biobank.

## Ethics Statement

The studies involving human participants were reviewed and approved by Mayo Clinic Individualized Medicine Biobank for Bipolar Disorder-IRBe 08-008794. The patients/participants provided their written informed consent to participate in this study.

## Author Contributions

NAN, BJC, MAF, and JMB conceptualized study design and drafted the manuscript. NAN, BJC, and CC analysis and interpretation of data. All authors critically revised the manuscript, provided critical revision, and important intellectual content to the article and approved the submitted version.

## Funding

This research was supported by Mayo Clinic benefactors and the Mayo Clinic Center for Individualized Medicine.

## Conflict of Interest

JV reports a grant-in-kind from Assurex, unrelated to the current study. PC has received research support from Neuronetics, Inc., NeoSync, Inc., and Pfizer. He has received grant-in-kind equipment and laboratory support for research studies from Assurex Health, Neuronetics, Inc. and MagVenture, Inc. He has served as a consultant for Myriad Neuroscience and Procter & Gamble. BS received grant support from Clinical and Translational Science CCaTS Small Grants Award, and Mayo Clinic. SM is or has been a consultant to or member of the scientific advisory boards of Avanir, Allergan now AbbVie, Bracket now Signant Health, Naurex, Idorsia, Intra-Cellular Therapies, Inc., Shire now Takeda, Sunovion, and Takeda. She is or has been a principal or co-investigator on studies sponsored by the Agency for Healthcare Research & Quality AHRQ, Avenir, AstraZeneca, Cephalon, Forest, Marriott Foundation, Medibio, National Institute of Mental Health, Orexigen Therapeutics, Inc., Jazz, Shire now Takeda, Sunovian, and Takeda Pharmaceutical Company Ltd. She is also an inventor on United States Patent No. 6,323,236 B2, Use of Sulfamate Derivatives for Treating Impulse Control Disorders, and along with the patent's assignee, University of Cincinnati, Cincinnati, Ohio, has received payments from Johnson & Johnson, which has exclusive rights under the patent. MF has received grant support from Assurex Health, Myriad, Pfizer, National Institute of Mental Health RO1 MH079261, National Institute of Alcohol Abuse and Alcoholism P20AA017830, Mayo Foundation; has been a consultant to Janssen Global Services, LLC, Mitsubishi Tanabe Pharma Corporation, Myriad, Sunovion, and Teva Pharmaceuticals; has received CME/Travel Support/presentation from CME Outfitters Inc. and Sunovian; Mayo Clinic has a financial interest in AssureRx and OneOme. The remaining authors declare that the research was conducted in the absence of any commercial or financial relationships that could be construed as a potential conflict of interest.

## Publisher's Note

All claims expressed in this article are solely those of the authors and do not necessarily represent those of their affiliated organizations, or those of the publisher, the editors and the reviewers. Any product that may be evaluated in this article, or claim that may be made by its manufacturer, is not guaranteed or endorsed by the publisher.
